# Feeding in Diphtheria

**Published:** 1901-06-22

**Authors:** 


### FEEDING IN DIPHTHERIA.

The feeding of patients suffering from diphtheria is often a matter of extreme difficulty, owing to the inability of so many of them to take or to retain nourishment given by the mouth. Among the difficulties in the way of feeding by the mouth are inability to swallow owing to pain or faucial swelling; regurgitation, partly due to paralysis, partly to swelling and interference with the proper co-ordination of the pharyngeal muscles; entrance of food into the larynx from the same causes; the exhaustion produced by feeding when this is resisted; and lastly, continued vomiting. Dr. R. G. Kirton,[1] of the Brook Fever Hospital, says that in the early stages of diphtheria milk should be the chief article of food. It may be mixed with some diluent such as barley-water, or given in the form of Benger's or Mellin's food. Children when they have the membrane on their throats will take nourishment fairly well, but when the membrane has peeled off they sometimes have difficulty and pain in swallowing, and require a lot of persuasion even to make them take a small quantity by the mouth. It is worthy of note that very few children are at first willing to drink out of a feeder. They much prefer a small medicine glass or a spoon. In regard to regurgitation, Dr. Kirton gives the very useful hint that all liquids should be thickened, milk being given as thick Benger's food, milk arrowroot, or milk jelly. Eggs may sometimes be tried, for if lightly boiled and made into a paste with breadcrumbs some patients suffering from regurgitation are able to swallow them. When nasal feeding has to be resorted to the patient should as a rule be fed every four hours. Milk, if necessary peptonised or as Benger's Food should be the chief ingredient, but raw meat juice, eggs, cream, brandy or medicines may be added as required. About four ounces is as much as a child from three to five years old will retain, and even this may have to be reduced. All foods should be strained, measured, and given warm. If vomiting be induced our next resource is rectal feeding, among other causes for the employment of which are epistaxis, or fright and struggling, caused by the passage of the nasal tube. Rectal feeding should be performed by means of a funnel and soft rubber tube, the latter being passed as high up the rectum as possible. The food should be allowed to flow in by force of gravity. A ball syringe should not be used for "it is impossible to gauge accurately the amount of pressure used" and "experience also shows that nutrients given this way are not so well retained." The amount to be given varies in young children from two to four ounces. Peptonised milk is the basis and often the sole constituent of food given in this way, but raw meat juice, white of eggs, and occasionally yolks of eggs are added. The bowel should be washed out every twenty-four hours and the stuff removed should be inspected so as to gain some idea as to whether the enemata are being absorbed or not. In some cases rectal feeding gives rise to diarrhœa, which is a serious matter, seeing that children sometimes have to depend upon this form of alimentation for long periods. When diarrhœa occurs the feeds should be at once diminished in quantity, or some ingredient should be omitted—for example, brandy, which not uncommonly causes irritation. Lastly, there is a mode of alimentation which must be borne in mind in cases in which it is impossible to introduce a sufficiency of food in other ways, namely, the subcutaneous injection of sterilised horse serum. From 20 to 40 cubic centimetres of this may be injected daily into the loose subcutaneous tissues of the trunk. The only indication against the use of these injections seems to be the occurrence of hæmorrhage into the subcutaneous tissues adjoining the site of the injection. It is impossible, as yet, to speak dogmatically of the utility of this measure, but Dr. Kirton says that his own impression is that it has been of value.

[1] Lancet, June 15.

